# Supplementary material for: Spin-orbit torque-driven skyrmion dynamics revealed by time-resolved X-ray microscopy
Source: Nat Commun. 2017 May 24;8:15573. doi: 10.1038/ncomms15573 (PMC5458085; doi:10.1038/ncomms15573)
Supplement: Supplementary Information — Supplementary Figures, Supplementary Notes and Supplementary References [file ncomms15573-s1.pdf]

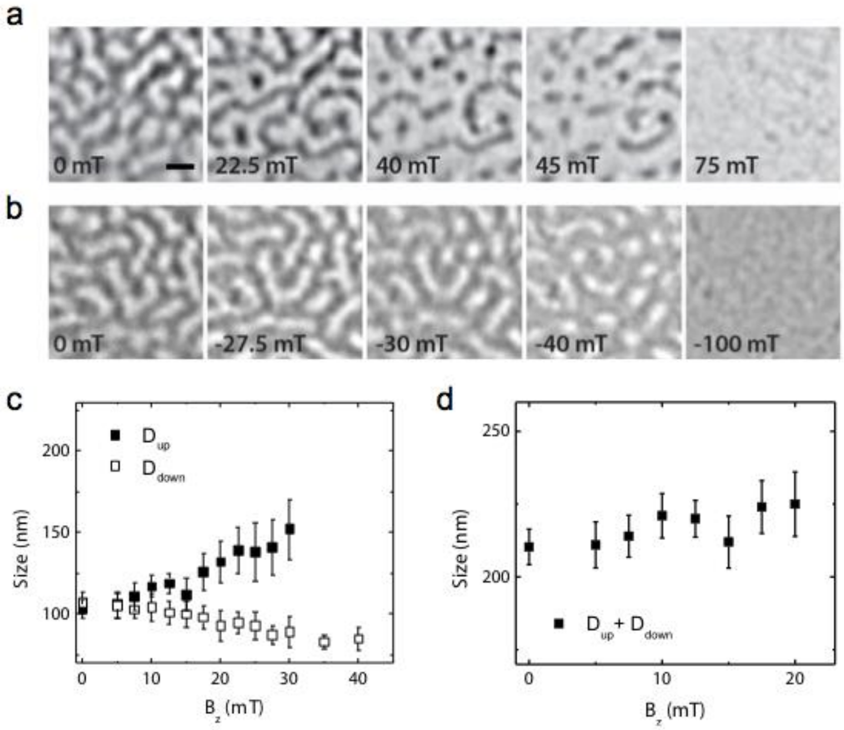

Supplementary Figure 1. Magnetic domain configuration under out-of-plane field application. (**a**), (**b)** MTXM images showing magnetic domain state acquired at a given out-of-plane magnetic field. Bright and dark contrasts correspond to up-oriented and down-oriented magnetic domains, respectively. Scale bar, 200 nm. (**c**) Domain width as a function of applied magnetic field for $B_z > 0$. The width of up-oriented domain increases while down-oriented domain shrinks to a finite terminal width ~ 90 nm. (**d**) Domain period (D_{up}+D_{down}) for relatively low field $B_z < 20$ mT. In this low field regime, the domain period is roughly constant ~ 215 nm. In the large field regime ($B_z > 20$ mT), the width definition of up-oriented domain becomes obscure as the labyrinth domains become connected and turn into an uniform magnetization state eventually. The error bards in (**c**) and (**d**) represent calculated standard deviations from 20 measurements.

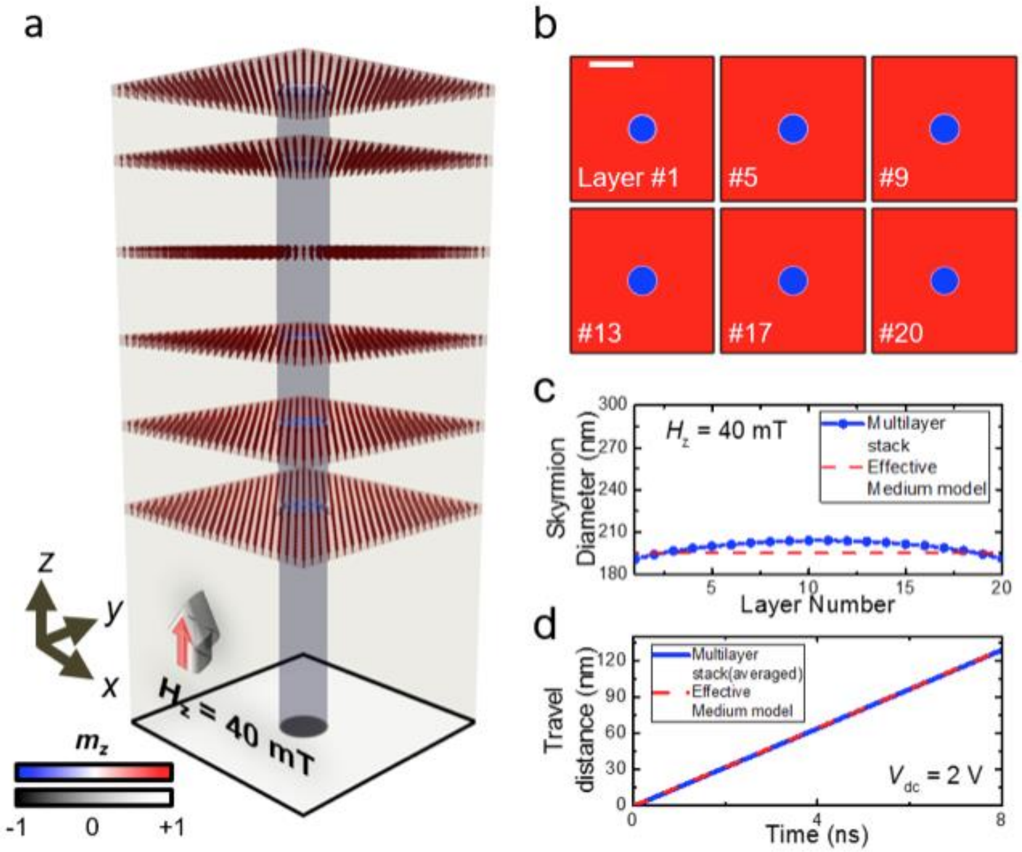

Supplementary Figure 2. Skyrmion structures in multilayer stack. (**a**) Illustration of a single skyrmion in Pt/CoFeB/MgO multilayer films stabilized by DMI and external magnetic filed, H_z = 30 mT, and its averaged out-of-plane magnetic contrast, which results in MTXM image. (**b**) Layer-resolved magnetic structures in 1st, 5th, 9th, 13th, 17th and 20th CoFeB layers in a 20-repeated multilayer stack. Scale bar, 300 nm. (**c**) Skyrmion diameter as a function of layer position. Skyrmion diameter calculated with the effective medium model is also shown (red dotted line). (**d**) The travel distance of a skyrmion in the presence of in-plane dc current flows under 2 V voltage application. The red and blue lines show the averaged travel distance with and without the effective medium model, respectively.

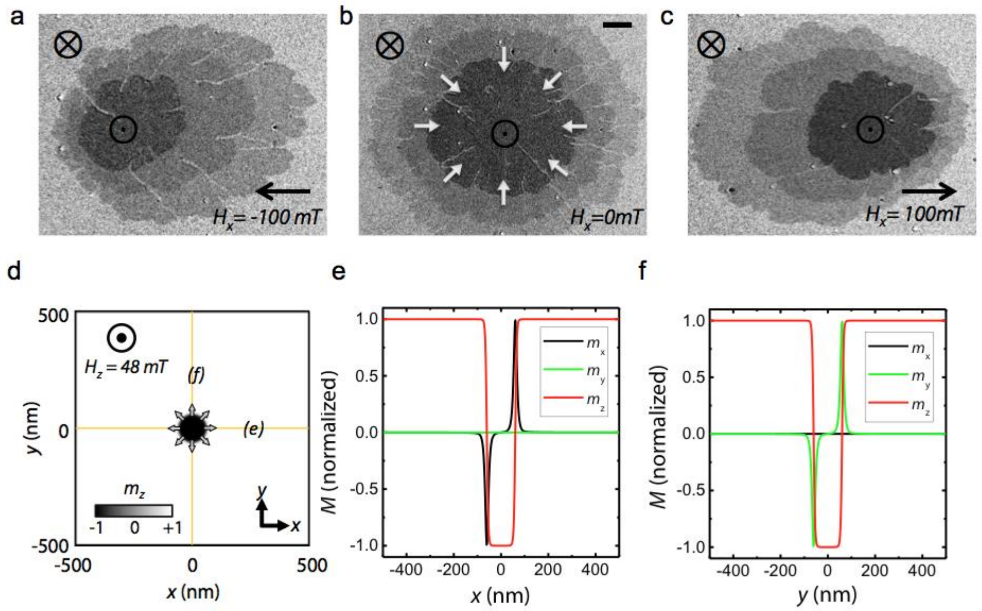

Supplementary Figure 3. Asymmetric domain expansion experiments and micromagnetic simulation on skyrmion profile. Up-oriented circular domain expansion in a Pt/CoFeB/MgO single layer film by an out-of-plane magnetic field (B_z) pulse in the presence of a constant in-plane magnetic field of (**a**) B_x=-100 mT, (**b**) B_x=0 mT and (**c**) B_x=100 mT. Scale bar, 25 μm. Each panel shows an overlap of three images, with each image acquired after applying an out-of-plane field-pulse. The domains expands symmetrically in all directions in the absence of an in-plane field as shown in (**b**) whereas asymmetric domain expansions are observed for H_x=-100 mT and 100 mT for (**a**) and (**c**), respectively. The position of the out-pointing arrows at the center of domains indicate the initial nucleation position of each circular domain. (**d**) Micromagnetic simulation snapshot after stabilizing a skyrmion at H_z=48 mT. Experimentally acquired material parameters were used to calculate the equilibrium magnetic state. Schematic arrows are included to indicate the direction of magnetic moments in the Néel DWs. Variation of magnetic moments, m_x, m_y and m_z, along (**e**) y=0 and (**f**) x=0 are plotted, confirming the left-handed Néel nature of a stabilized skyrmion.

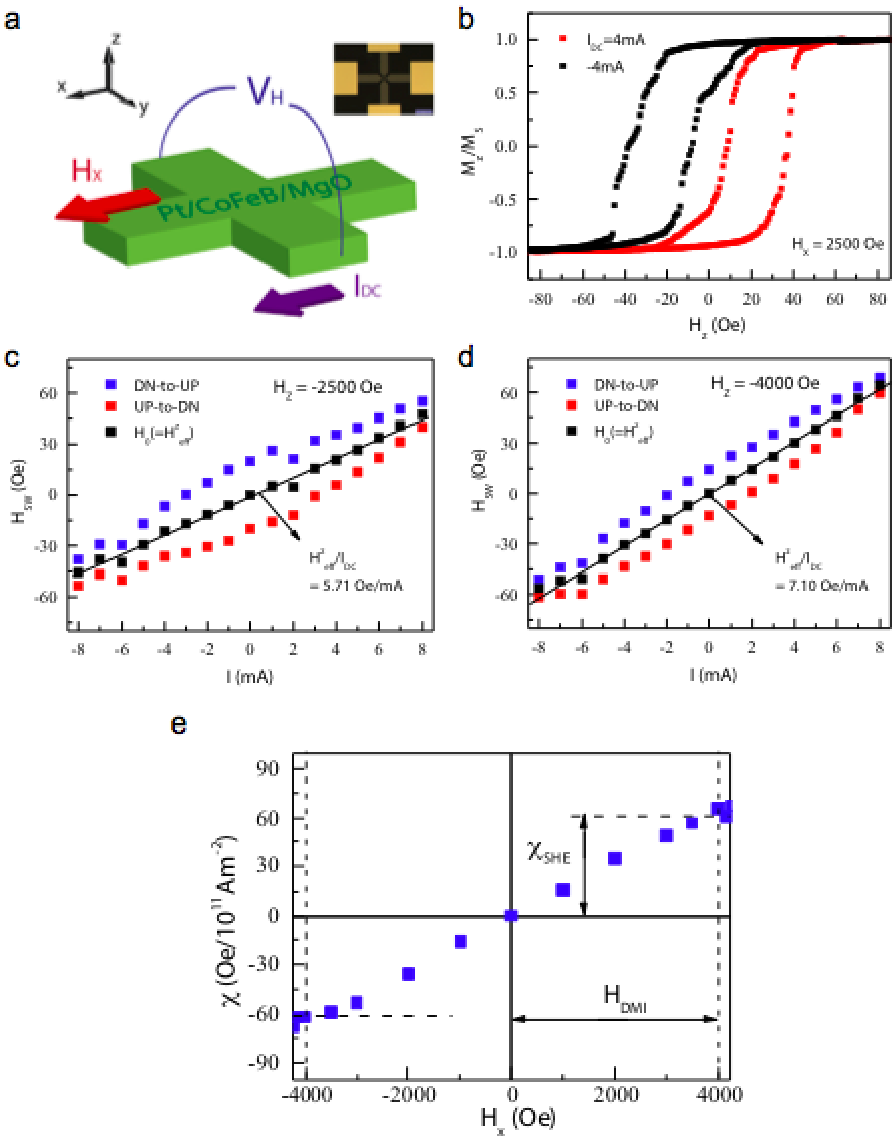

Supplementary Figure 4. Electrical measurement of spin-Hall effect efficiency and DMI constant. (**a**) Schematic of the Hall bar device and measurement scheme. In-plane magnetic field and dc-current are applied while the anomalous Hall voltage measurement is conducted. The inset shows scanning electron microscope image of an actual device used for measurement. (**b**) AH loops under dc-currents I_{dc} = ±4 mA and an in-plane field H_x = 2500 Oe. (**c**), (**d**) Switching fields H_{SW} for down-to-up (blue square) and up-to-down (red square) magnetization reversals at H_x = -2500 Oe and H_x = -4000 Oe, respectively. The center of hysteresis loop H_0, which is the net effective spin-Hall field in the *z* direction, is shown to more clearly show the amount of AH loop shift. For each figure, the spin-Hall effective field per unit current in mA is calculated and shown. (**e**) The measured effective χ (= $\chi_{SHE}\cos\Phi$) as a function of H_x. The measure of effective spin-Hall efficiency, χ_{SHE}, and an estimate of DMI effective field, H_{DMI}, is indicated.

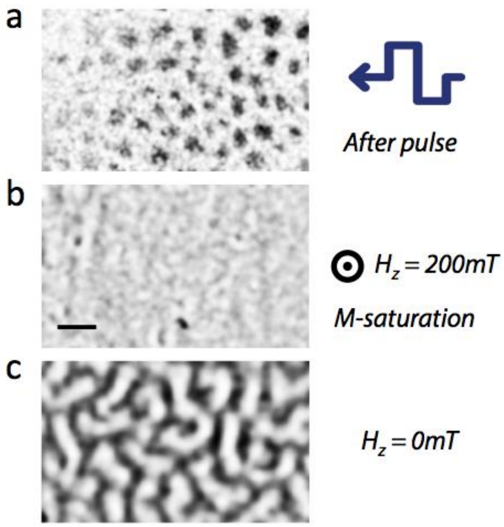

Supplementary Figure 5. Effect of pulse application on the magnetic property of sample. (**a**) A MTXM image acquired after applying transforming domain phase from labyrinth state to multiple skyrmionic state after the application of bipolar pulses at $B_z = 0$ mT (adopted from Fig. 2 in main text). Bipolar pulse trains are injected at f=3.33 MHz for 5 s. MTXM images after applying saturation magnetic field, H_z=200 mT, and turning off the field application are shown in (**b**) and (**c**), respectively. Scale bar, 200 nm.

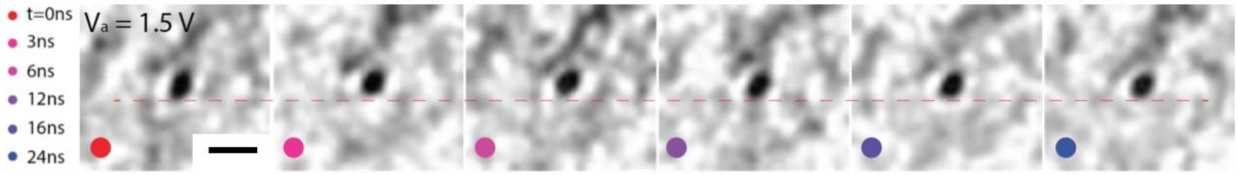

Supplementary Figure 6. Dynamic behavior of a magnetic skyrmion when V_a=1.5 V. Magnetic skyrmion configuration at different time delays for voltage amplitudes of V_a=1.5 V. The same pulse profile, shown in Fig. 3b in main text, is used. The coloured circles indicate the time delay. Scale bar, 200 nm.

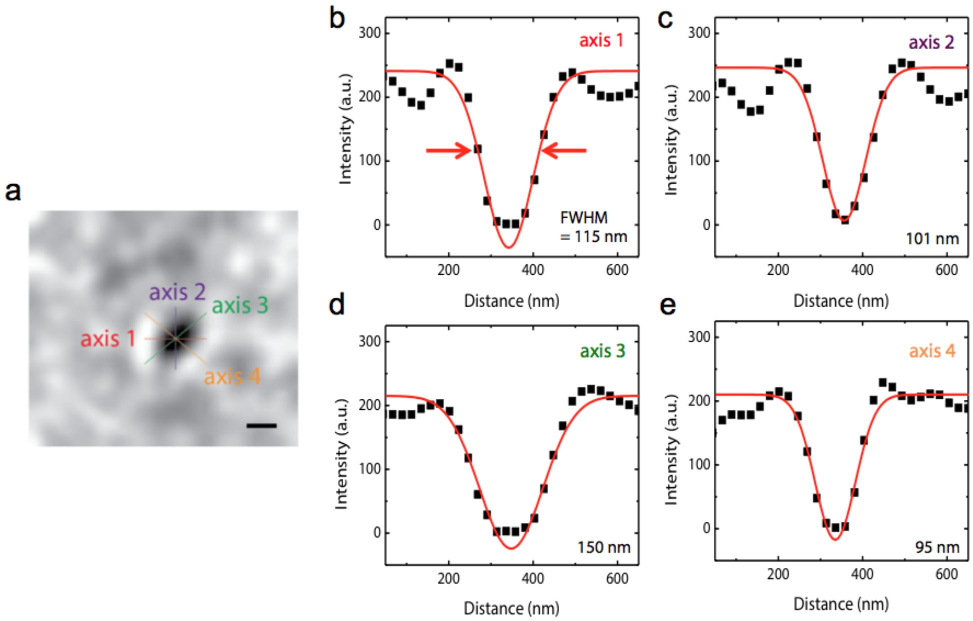

Supplementary Figure 7. Determination of skyrmion diameter. (**a**) A representative MTXM image in Fig. 3d in the main text. A single skyrmion with four lines crossing the center of the skyrmion. Lines are drawn along the direction *y*=0 (axis 1), *x*=0 (axis 2), *y*=*x* (axis 3) and *y*=-*x* (axis 4) and used for contrast line-scans. Scale bar, 100 nm. (**b**)-(**e**) The contrast line scans with its Gaussian fitting and FWHM, which is used to determine the diameter of skyrmions.

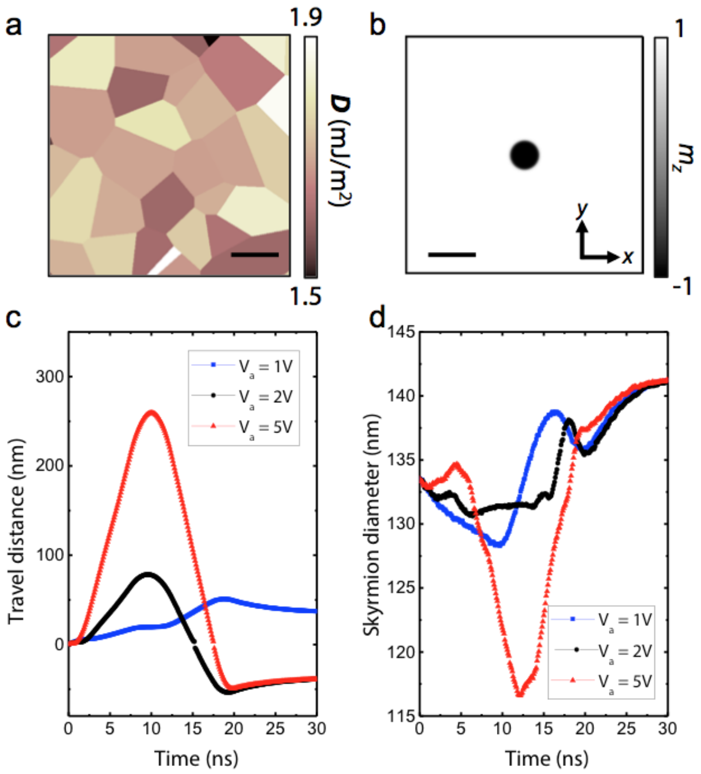

Supplementary Figure 8. Skyrmion dynamis in a ferromagnetic film with non-uniform DMI constant. (**a**) A simulated magnetic mesh and the distribution of DMI constant in the film. DMI randomly varies between 1.5 mJ m^{-2} and 1.9 mJ m^{-2}. (**b**) Initial skyrmion state at equilibrium state. External field of B_z=48 mT is applied to stabilize a skyrmion. (**c**) Skyrmion travel distance and (**d**) skyrmion dimater as a function of pulse-time. For skyrmion travel distance, initial skyrmion position was set to d=0, so the negative distance exhibits that skyrmion moved beyond its initial position. The sample pulse profile shown in Fig. 4 in main text is used for V_a=1 V (blue), V_a=2 V (black) and V_a=5 V (red), respectively. Scale bars, 200 nm.

Supplementary Note 1. DMI measurement from the field-driven domain width variation

We measured the domain width from the full-field magnetic transmission X-ray microscopy (MTXM) images as the external magnetic field is changed. The domain width reflects the competition between the demagnetizing energy and the domain wall (DW) energy: the demagnetizing energy prefers DW generation whereas there is an accompanying energy cost for the generation of each DW. However, strong Dzyaloshinskii-Moriya interaction (DMI) can effectively reduce the DW energy cost by locally stabilizing Néel walls. In the presence of DMI, the DW energy is given as $\sigma_{\mathrm{DW}} = 4\sqrt{AK_{\mathrm{u,eff}}} - \pi|D|$, where A is the exchange stiffness, $K_{\mathrm{u,eff}}$ is the effective uniaxial anisotropy constant, and D is the DMI constant[1,2]. Therefore, by calculating the DW surface energy from domain widths, we can determine the DMI constant analytically.[3]

Supplementary Fig. 1a and b show the variation of domain configuration as a function of external magnetic field. When B_z=0, up- and down-domains have equal widths, and increasing or decreasing the magnetic field drives the net magnetization to $+M_S$ or $-M_S$, respectively. From the high-resolution (~25 nm spatial resolution) MTXM image at a specific B_z, the width of up/down domains were measured and plotted in Supplementary Fig. 1c for the case of $B_z > 0$. As B_z increases, the up-domain expands and the down-domain shrinks as expected. For relatively low field $B_z < 20$ mT, the widths of up/down domains changes while maintaining a constant domain period, $D_{\mathrm{up}} + D_{\mathrm{down}}$, which is measured to be ~ 215 nm in Supplementary Fig. 1d. For higher fields $B_z > 20$ mT, the width of the down-domain approaches a finite value ~85 nm and the length of the domains decrease, eventually disappearing at a saturation field. On the other hand, as seen by large error bars of D_{up} for $B_z > 20$ mT in Supplementary Fig. 1c, it becomes obscure to measure the width of the up-domain precisely for higher field because the labyrinth domain state transforms to a uniformly magnetized domain state. Using the domain period at low field (D_p =

D_{up} + D_{down}), we can calculate the DW surface energy using Eq. (1), while the same can be done with the high-field terminal down domain width using Eq. (2)[4],

$$\frac{\sigma_{\mathrm{DW}}}{\mu_0 M_{\mathrm{s}}^2 t} = \frac{D_{\mathrm{p}}^2}{t^2} \sum_{odd\ n=1}^{\infty} \frac{1}{(\pi n)^3} \left[1 - \left(1 + 2\pi n t / D_{\mathrm{p}}\right) \exp\left(-2\pi n t / D_{\mathrm{p}}\right) \right] \tag{1}$$

$$\frac{2\pi \sigma_{\mathrm{DW}}}{\mu_0 M_{\mathrm{s}}^2 t} = \ln[1 + (d_{\downarrow,\mathrm{min}} / t)^2] + (d_{\downarrow,\mathrm{min}} / t)^2 \ln[1 + (d_{\downarrow,\mathrm{min}} / t)^{-2}] \tag{2}$$

Using material parameters measured from the VSM measurements and the relation between domain wall surface energy and DMI constant, $\sigma_{\mathrm{DW}} = 4\sqrt{AK_{\mathrm{u,eff}}} - \pi|D|$, Eq. (1) and (2) yields the DMI constants of $|D|$=1.66 mJ m^{-2} and $|D|$=1.35 mJ m^{-2}, respectively. These DMI constants are in good agreement with the DMI constant measured in the Pt/CoFeB/MgO structure[5]. The DMI constant is also acquired by using a different method following Supplementary Ref. 5, which is described in Supplementary Note 4. The DMI constant reported in the main text, $|D|$=1.68 mJ m^{-2}, is the mean of these three values.

Supplementary Note 2. Magneto-static coupling of the magnetic layers in the Pt/CoFeB/MgO multilayers

Using full-stack micromagnetic simulations, not the effective medium model, we examined magnetic structures in each CoFeB layer in a 20-times-repeated [Pt (3 nm) / $Co_4Fe_4B_2$ (0.8 nm) / MgO (1.8 nm)]$_{20}$ multilayer stack with the cell size of 2×2×0.8 nm^2. Material parameters and details on the simulations are explained in Methods. We applied an external bias B_z=+40 mT to initialize a single magnetic skyrmion in the main text. Supplementary Fig. 2b shows magnetic structures in 1st, 5th, 9th, 13th, 17th and 20th CoFeB layers, indicating that all layers are magneto-statically coupled and exhibit the same magnetic structure, a single skyrmion in this case. Supplementary Fig. 2c shows a skyrmion diameter as a function of the layer position. It is noticeable that skyrmion diameter is not the same for each magnetic layer, mainly due to non-uniform out-of-plane demagnetizing field. Because we can only observe average-diameter of skyrmions in MTXM measurement, this implies that the experimentally measured skyrmion sizes (see Supplementary Note 7) have an error range of approximately ~10 %. However, as simultaneously shown in Supplementary Fig. 2c, it should be noted that the effect of the non-uniform skyrmion diameter across the entire stack is almost ignorable when we compare it to the effective medium model. Moreover, we reveal in Supplementary Fig. 2d that the dc current-driven skyrmion dynamics acquired from two models agree very well. Therefore, we only used the effective medium model to simulate the dynamic behaviors of a skyrmion in the main text to reduce the effective calculation time.

Supplementary Note 3. Out-of-plane domain expansion under a constant in-plane field in a single Pt/CoFeB/MgO layer

Here we image the out-of-plane domain expansion in a single Pt/CoFeB/MgO continuous film under constant in-plane field application using a previously established technique[6,7], in order to determine the nature of the DWs in the structure. This measurement was conducted by wide-field magneto-optical Kerr effect (MOKE) microscopy, which effectively measures the magnetic contrast in a continuous film by using a polarized light microscope and a CCD camera.

Supplementary Fig. 3 shows representative MOKE images of domain expansion in a single Pt/CoFeB/MgO layer under H_x field application. It has been proved that magnetic domains enclosed by chiral Néel DWs show asymmetric out-of-plane field driven domain expansion in the presence of a constant in-plane field since the applied in-plane field is either parallel or antiparallel to a Néel DW[6,7]. A parallel H_x field can further stabilize Néel DWs causing faster DW displacement while an antiparallel field induces slower DW motion by destabilizing it. Supplementary Fig. 3b shows the expansion of a circular domain in the absence of an in-plane field, which shows symmetric domain expansion in all directions. When an in-plane field is applied either along the $+x$ or $-x$ direction, the domain expands asymmetrically as shown in Supplementary Fig. 3a and 3c, due to the chiral nature of Néel DWs enclosing it. This measurement allows us to determine the direction of magnetic moments within the Néel walls as shown schematically in Supplementary Fig. 3b. To further confirm the chiral nature of domain walls, we performed micromagnetic simulations using experimentally obtained material parameters shown in the main text. Supplementary Fig. 3d first shows a snapshot of a skyrmion at an equilibrium state in the presence of external magnetic field, H_z=48 mT, acquired from simulation. At this skyrmion state, we plotted the variation of magnetic moments, m_x, m_y and m_z,

along two lines: y=0 (x-axis) and x=0 (y-axis) as shown in Supplementary Fig. 3e and f, respectively. At y=0, only x-component magnetization, m_x, varies while y-component magnetization, m_y, remains the same, and vice versa. The simulated magnetization profile perfectly matches with experimental observation shown in Supplementary Fig. 3a-c. These correspond to a left-handed chirality of the Néel DWs and agrees well with other Pt-interfaced structures previously reported[3,8]. This chirality measurement implies that the magnetic skyrmions presented in the main text are indeed hedgehog-type chiral magnetic skyrmions with Néel DWs[6,7]. The left-handed chirality is also inferred from the direction of spin Hall effect (SHE)-driven translation of skyrmions in the main text Fig. 3e.

Supplementary Note 4. Determination of spin Hall torque efficiency and DMI constant in the Pt/CoFeB/MgO structure

Here we measure the spin-Hall torque efficiency, χ_{SHE}, in a single Pt/CoFeB/MgO layer using an electrical measurement technique developed by Pai *et al.*, in Supplementary Ref. 5. In this measurement scheme, we measure the H_z-driven out-of-plane hysteresis loops using anomalous Hall (AH) voltage measurement under the additional application of an in-plane field H_x and an in-plane dc-current I_{DC}. The application of H_x and I_{DC} causes the shift of the hysteresis loop, and this current-induced shift can be used to quantify χ_{SHE} while providing an estimate of the DMI effective field H_{DMI}.

In the presence of SHE and DMI[2,8], the effective field acting upon a Néel-type DW can be expressed as $H^z_{eff} = \chi_{SHE} \cos\Phi \times J_e$ with χ_{SHE} as the SHE efficiency, Φ as the angle between spin moment in DWs and *x*-axis, and J_e as the in-plane current density. In the case of homochiral Néel DWs, the SHE-induced Slonczewski-like SOT can *drive* Néel DWs because of the opposite signs of H^z_{eff} for up-down (Φ=180°) and down-up (Φ=0°) domains. However, in the presence of a strong in-plane field where the field can overcome the effective DMI-field in a Néel wall and reverse the magnetic moment, the SHE-induced torque can expand or contract domains. In this measurement, therefore, the hysteresis loop shift can be observed when an in-plane field/current are simultaneously applied because the in-plane field can break the symmetry between up-to-down and down-to-up Néel DWs, and the in-plane current acts differently on two DWs. Without an in-plane field, only a small reduction in coercive field occurs mainly due to the Joule-heating while the center of hysteresis loop remains at the same position[5].

Supplementary Fig. 4 shows the result of the spin-Hall efficiency and DMI effective field measurements. As schematically shown in Supplementary Fig. 4a, we measured the AH voltage

while sweeping the out-of-plane magnetic field in the presence of an in-plane magnetic field and in-plane dc-current. The AH measurements were conducted at various in-plane dc currents (I_{dc}) and in-plane fields (H_x) to characterize magnetization switching as a function of I_{dc} and H_x. The in-plane field was applied along the x-axis due to chiral Néel DWs in this geometry points toward either $+x$ or $-x$ direction. Representative normalized AH loops with H_x = 2500 Oe and I_{dc}=±4 mA are shown in Supplementary Fig. 4b. The AH loops along the H_z axis are oppositely shifted for I_{dc}=+4 mA and I_{dc}=-4 mA, and this implies the existence of current-induced effective field H^z_{eff} due to a SHE-induced Slonzewski-like torque. We then plotted switching fields for both up-to-down and down-to-up magnetization reversals for two representative in-plane fields of H_x=-2500 Oe and H_x=-4000 Oe in Supplementary Fig. 4c and d, respectively. In each plot, the shifted center positions of AH loop, H_0, are plotted and the shifts offer the direct measure of effective field to z-axis at a given H_x and I_{dc}. Each plot also provides an estimate of H^z_{eff}/I_{dc}, which is the effective SHE-induced field per unit current in mA unit. By plotting H^z_{eff} versus I_{dc} for a wide range of in-plane fields H_x and converting the current into current density by considering device geometry, we can finally plot the efficiency $\chi(=H^z_{eff}/J_e)$ as a function of in-plane field H_x as shown in Supplementary Fig. 4e. The efficiency χ increases linearly with H_x up to H_x=±4000 Oe, where it saturates. This observation is consistent with the report that an average of mangetic moments in Néel wall changes from <cosΦ = 0> (equal number of DWs antiparallel or parallel to H_x) to <cosΦ = 1> (all DWs parallel to H_x) when H_x approaches to H_{DMI}[2]. From this model, we find that SHE-induced effective field and an estimate of effective DMI field are χ_{SHE}=62 Oe/10^{11} A m^{-2} and $|H_{DMI}|$ = 4000 Oe, respectively, in our Pt/CoFeB/MgO structure.

We can further calculate the effective spin-Hall angle and DMI constant from the measured χ_{SHE} and $|H_{DMI}|$. χ_{SHE} can be expressed as $\chi_{SHE} = (\pi/2)(\hbar\theta/2e\mu_0 M_S t_{FM})$, where θ is

the effective spin-Hall angle, M_S is the saturation magnetization and t_{FM} is the thickness of ferromagnetic layer. The magnitude of DMI can also be calculated from the measured $|H_{DMI}|$ by using $|D| = \mu_0 M_S \Delta |H_{DMI}|$ in Supplementary Ref. 2, where Δ is the domain width defined by $\Delta = \sqrt{A / K_{u,eff}}$. By using material parameters obtained from VSM, we can determine that the effective spin-Hall angle in our film is θ= +0.08 and the DMI constant is $|D|$ = 2.02 mJ m^{-2}. The sign and magnitude of spin-Hall angle are in good agreement with other reported values using Pt-interfaced films in Supplementary Ref. [8,9].

Supplementary Note 5. Magnetic domain configuration after pulse applications

In Fig. 2 of the main text, we showed that the application of consecutive bipolar pulses could transform the labyrinth domains into multiple skyrmions. However, it is also important to show that the transformation is not from the current-induced sample property variation, which might be possible when the electric currents are large enough to cause significant Joule-heating. Large Joule-heating could lead to structural changes that would alter magnetic properties even after the current pulse is turned off. To investigate the effect, we have swept an external field after the pulse application and obtained magnetic domain images. Supplementary Fig. 5 shows MTXM images after pulse-induced skyrmion generation (Supplementary Fig. 5a), saturation field application (Supplementary Fig. 5b) and turning-off the external field (Supplementary Fig. 5c). When a large external field, H_z=200 mT, was applied in Supplementary Fig. 5b, most of created skyrmions were annihilated, leading to a uniform magnetization state. We then turned off the external field and it drove the magnetization state to the labyrinth state as shown in Supplementary Fig. 5c, and this confirms that the magnetic property has not been changed due to bipolar current pulses. We believe this simple observation implies that the pulse-induced skyrmion generation is indeed from the strong topological fluctuation induced by oscillating spin currents.

Supplementary Note 6. Skyrmion dynamics at V_a=1.5V

Here we show experimentally observed skyrmion dynamics at the pulse amplitude of V_a=1.5 V. In Fig. 3 in main text, we show skyrmion dynamics when the bipolar pulse amplitudes are V_a=2 V and V_a=2.5 V, where the pulses induce skyrmion excitation behaviours. However, when the pulse amplitude is relatively small, as shown in Supplementary Fig. 6, there is no observable shape-wise transformation. This indicates that the external current pulse amplitude should be larger than j_{th} to induce the excitation bahaviors, and the current threshold for our sample is roughly $|j_{th}| = 1 \times 10^{11}$ A m^{-2}.

Supplementary Note 7. Determination of the skyrmion diameter from MTXM images

Here we explain the skyrmion diameter determination procedure used to extract the actual diameter of the individual skyrmion from MTXM images. The diameter was determined by fitting four line-scans of the XMCD signal across the MTXM image as indicated in Supplementary Fig. 7. Each line-scan was fitted to a Gaussian, and the full-width at half maximum (FWHM) was used to determine the skyrmion diameter along the given axis. The diameter used in the main text was calculated by $D_{\mathrm{skyrmion}} = \sqrt{(D_{\mathrm{y=0}}^2 + D_{\mathrm{x=0}}^2 + D_{\mathrm{y=x}}^2 + D_{\mathrm{y=-x}}^2)/4}$, and the diameter of the represented skyrmion in Supplementary Fig. 7 was measured to be ~117 nm using this formula. The skyrmion diameters and domain widths mentioned in the main text were calculated following this method.

Supplementary Note 8. Effect of non-uniform DMI constant on skyrmion dynamics

In the main text, we first generated multiple magnetic skyrmions electrically, and then isolated a single skyrmion for dynamics measurement by applying external magnetic field. Because one might think that the isolated skyrmion is a pinned skyrmion, in this note, we show how a skyrmion behaves in the presence of skyrmion pinning sites. In a previous study by Woo *et al.*[3], it was shown that the pinned skyrmions annihilate rather easily by external magnetic field or electric current. Because a local variation in interface-induced DMI is likely responsible, they show that skyrmions pinned at a region with locally reduced DMI has a lower energy barrier for annihilation. Analogously, we believe that our studied skyrmion, which survived even at a high background magnetic field, has more ideal internal magnetic structure resembling an ideal skyrmion structure with the largest local DMI. To reveal the effect of skyrmion pinning on its dynamic behaviors, we have conducted additional micromagnetic simulations shown in Supplementary Fig. 8.

Supplementary Fig. 8 shows the effect of non-uniform DMI constant on skyrmion dynamics: i) displacement and ii) size variation. Supplementary Fig. 8a first shows the simulated distribution of DMI constant, which randomly varies between 1.5 mJ m^{-2} and 1.9 mJ m^{-2} across a simulation mesh. Note that the variation is only ~20 % with respect to the measured average DMI value, 1.68 mJ m^{-2}. We employed *random* DMI distribution, which is the likely case in a real sample. We first created a single skyrmion and the skyrmion found its stabilized position and size in the given potential landscape. To mimic the experimental studies shown in Fig. 4 of the main text, we applied bipolar current pulses (with the same profile shown in Fig. 4b of the main text) and examined the displacement and size variation of a skyrmion by varying the pulse amplitude from 1 V to 5 V. Supplementary Fig. 8c and d reveal that, regardless

of pulse amplitude, the skyrmion behaves in a very random fashion in the presence of DMI pinning. More importantly, for both travel distance and size variation, the skyrmion never came back to its original state.

Note that the stroboscopic pump-probe technique of MTXM restricts the imaging to fully reproducible magnetic events by synchronizing the incoming X-ray photon flashes (probe) and injecting current pulses (pump). This analysis reveals that, if observed skyrmion was at or near DMI pinning sites, we could not observe its dynamical behavior using stroboscopic time-resolved X-ray measurement. Thus, we believe that the observed skyrmion in main text was positioning at a region where the interfacial DMI is quite uniform, where the variation is significantly lower than 20%. It also agrees well with previous report, revealing the low-pinning characteristic of the amorphous-CoFeB based ferromagnetic multilayers.[3]

Supplementary References

1. Heide, M., Bihlmayer, G. & Blügel, S. Dzyaloshinskii-Moriya interaction accounting for the orientation of magnetic domains in ultrathin films: Fe/W(110). *Phys. Rev. B* **78,** 140403 (2008).
2. Thiaville, A., Rohart, S., Jué, É., Cros, V. & Fert, A. Dynamics of Dzyaloshinskii domain walls in ultrathin magnetic films. *EPL Europhys. Lett.* **100,** 57002 (2012).
3. Woo, S. *et al.* Observation of room-temperature magnetic skyrmions and their current-driven dynamics in ultrathin metallic ferromagnets. *Nat. Mater.* **15,** 501–506 (2016).
4. Johansen, T. H., Pan, A. V. & Galperin, Y. M. Exact asymptotic behavior of magnetic stripe domain arrays. *Phys. Rev. B* **87,** 060402 (2013).
5. Pai, C.-F., Mann, M., Tan, A. J. & Beach, G. S. D. Determination of spin torque efficiencies in heterostructures with perpendicular magnetic anisotropy. *Phys. Rev. B* **93,** 144409 (2016).
6. Je, S.-G. *et al.* Asymmetric magnetic domain-wall motion by the Dzyaloshinskii-Moriya interaction. *Phys. Rev. B* **88,** 214401 (2013).
7. Hrabec, A. *et al.* Measuring and tailoring the Dzyaloshinskii-Moriya interaction in perpendicularly magnetized thin films. *Phys. Rev. B* **90,** 020402 (2014).
8. Emori, S., Bauer, U., Ahn, S.-M., Martinez, E. & Beach, G. S. D. Current-driven dynamics of chiral ferromagnetic domain walls. *Nat. Mater.* **12,** 611–616 (2013).
9. Liu, L., Lee, O. J., Gudmundsen, T. J., Ralph, D. C. & Buhrman, R. A. Current-Induced Switching of Perpendicularly Magnetized Magnetic Layers Using Spin Torque from the Spin Hall Effect. *Phys. Rev. Lett.* **109,** 096602 (2012).
